# SARS-CoV-2 suppresses TLR4-induced immunity by dendritic cells via C-type lectin receptor DC-SIGN

**DOI:** 10.1371/journal.ppat.1011735

**Published:** 2023-10-16

**Authors:** Lieve E. H. van der Donk, Marta Bermejo-Jambrina, John L. van Hamme, Mette M. W. Volkers, Ad C. van Nuenen, Neeltje A. Kootstra, Teunis B. H. Geijtenbeek

**Affiliations:** 1 Department of Experimental Immunology, Amsterdam UMC location University of Amsterdam, Amsterdam, The Netherlands; 2 Amsterdam institute for Infection and Immunity, Infectious Diseases, Amsterdam, The Netherlands; 3 Institute of Hygiene and Medical Microbiology, Medical University of Innsbruck, Innsbruck, Austria; The Ohio State University, UNITED STATES

## Abstract

SARS-CoV-2 causes COVID-19, an infectious disease with symptoms ranging from a mild cold to severe pneumonia, inflammation, and even death. Although strong inflammatory responses are a major factor in causing morbidity and mortality, superinfections with bacteria during severe COVID-19 often cause pneumonia, bacteremia and sepsis. Aberrant immune responses might underlie increased sensitivity to bacteria during COVID-19 but the mechanisms remain unclear. Here we investigated whether SARS-CoV-2 directly suppresses immune responses to bacteria. We studied the functionality of human dendritic cells (DCs) towards a variety of bacterial triggers after exposure to SARS-CoV-2 Spike (S) protein and SARS-CoV-2 primary isolate (hCoV-19/Italy). Notably, pre-exposure of DCs to either SARS-CoV-2 S protein or a SARS-CoV-2 isolate led to reduced type I interferon (IFN) and cytokine responses in response to Toll-like receptor (TLR)4 agonist lipopolysaccharide (LPS), whereas other TLR agonists were not affected. SARS-CoV-2 S protein interacted with the C-type lectin receptor DC-SIGN and, notably, blocking DC-SIGN with antibodies restored type I IFN and cytokine responses to LPS. Moreover, blocking the kinase Raf-1 by a small molecule inhibitor restored immune responses to LPS. These results suggest that SARS-CoV-2 modulates DC function upon TLR4 triggering via DC-SIGN-induced Raf-1 pathway. These data imply that SARS-CoV-2 actively suppresses DC function via DC-SIGN, which might account for the higher mortality rates observed in patients with COVID-19 and bacterial superinfections.

## Author summary

Superinfections with bacteria during severe COVID-19 lead to worse prognosis of COVID-19 patients. Here we have identified a novel mechanism triggered by SARS-CoV-2 that suppresses immune responses to bacteria. We found that exposure of human dendritic cells (DCs) to SARS-CoV-2 or the Spike glycoprotein blocks TLR4-induced immune responses. Our data strongly suggest that SARS-CoV-2 interacts with the C-type lectin receptor DC-SIGN, which blocks TLR4-induced immunity. Taken together, our data suggest that SARS-CoV-2 actively suppresses DC function via DC-SIGN, which might account for the higher mortality rates observed in patients with COVID-19 and bacterial superinfections.

## Introduction

SARS-CoV-2 causes coronavirus disease 2019 (COVID-19), which is an infectious disease characterized by strong induction of inflammatory cytokines, progressive lung inflammation and potentially multi-organ dysfunction [1–3]. SARS-CoV-2 infects epithelial cells of the airways using the receptor angiotensin-converting enzyme 2 (ACE2) for infection [4,5]. Notably, it has been reported that COVID-19 patients, in particular severely ill patients, are vulnerable to viral, fungal or bacterial superinfections [6–10]. Superinfections arise when a primary infection is followed by a secondary infection [11]. Many of the superinfections in COVID-19 patients are caused by bacteria, for instance through hospital-acquired pneumonia or ventilator-acquired pneumonia with different virulent bacterial species such as *Pseudomonas (P*.*) aeruginosa* or *Klebsiella (K*.*) pneumoniae* [6–10,12]. Severe COVID-19 with superinfections is therefore associated with significantly worse prognosis [10,12]. However, it is currently unclear whether increased susceptibility of COVID-19 patients to bacterial superinfections is due to systemic inflammation or SARS-CoV-2 specifically affecting defense against bacterial infections.

Dendritic cells (DCs) are located throughout the mucosal barrier tissues such as the airways and lungs, and are essential for defense against infections by microbes including bacteria and viruses. DCs sense foreign microbes with pattern recognition receptors (PRRs), leading to antigen presentation to T cells and potent adaptive immune responses [13]. Toll-like receptors (TLR) are important PRRs for sensing bacteria and TLR triggering induces type I Interferon (IFN) and proinflammatory cytokine responses, required for adaptive immunity [14]. The TLR family member TLR4 is highly expressed by DCs and senses the bacterial component lipopolysaccharide (LPS) [15]. Notably, whereas recent research has focused on TLR4-mediated immune activation by SARS-CoV-2 [16–19], the suppressive effects of SARS-CoV-2 on the immune response are not yet investigated.

Here, we investigated whether SARS-CoV-2 affects DC-induced immunity to bacteria using both SARS-CoV-2 S protein and SARS-CoV-2 primary isolate (hCoV-19/Italy). Notably, our data strongly suggest that SARS-CoV-2 suppresses DC-induced immune responses by TLR4, whilst signaling through other TLRs was not affected. Our data suggest that SARS-CoV-2 S protein as well as SARS-CoV-2 virus particles bind DC-SIGN, which induces signaling via kinase Raf-1 suppressing TLR4 signaling. Thus, we have identified a novel mechanism of immunosuppression by SARS-CoV-2 that might underlie the increased susceptibility to Gram-negative bacteria and targeting this pathway might attenuate bacterial infections during COVID-19.

## Results

### SARS-CoV-2 S protein specifically suppresses TLR4 activation

**Fig 1:**
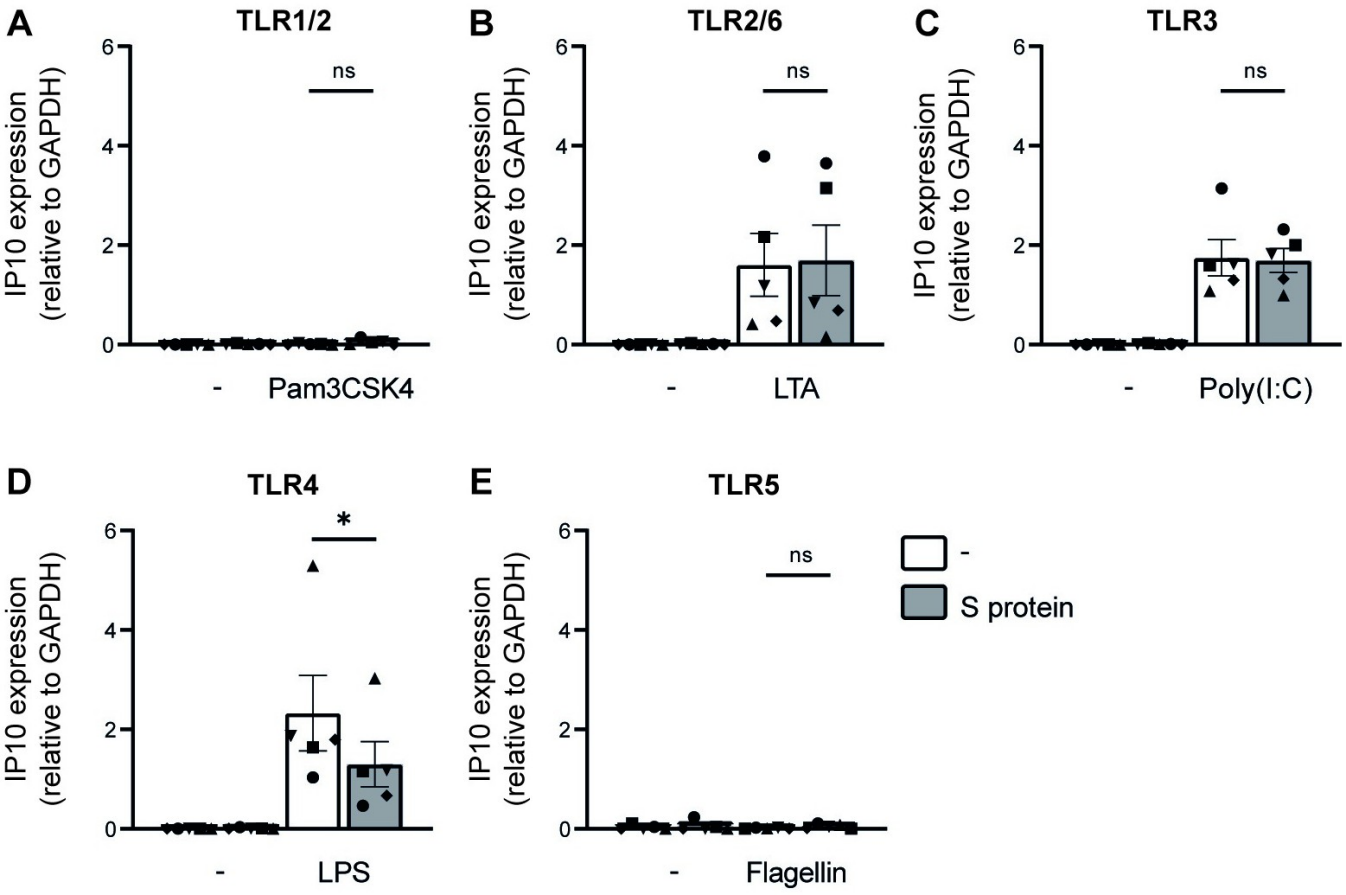
S protein modulates TLR4 signaling. (A-E) DCs were pre-incubated with S protein before exposure to a plethora of bacterial TLR stimuli. After 6h incubation, cells were lysed and mRNA levels of IP10 were determined by qPCR. Data show the mean values and SEM. Statistical analysis was performed using student’s *t*-test. Data represent n = 5 DC donors obtained in three separate experiments with each symbol representing a different donor. *p<0.05; ns = non-significant.

To investigate whether SARS-CoV-2 affects DC function towards other external stimuli, we exposed DCs to recombinant SARS-CoV-2 S protein before adding different TLR agonists, and screened for immune responses by measuring induction of interferon (IFN)-stimulated gene (ISG) IP10. As we have previously shown, S protein alone did not induce DC activation [19]. TLR agonists against TLR2/6, TLR3 and TLR4 induced IP10 (Fig 1), while TLR1/2 and TLR5 agonists did not. Notably, pre-incubation with S protein decreased induction of IP10 by the TLR4 agonist, but not by the other TLR agonists. These data suggest that SARS-CoV-2 specifically modulates TLR4 signaling.

### SARS-CoV-2 S protein is involved in the suppression of immune responses by DCs

**Fig 2:**
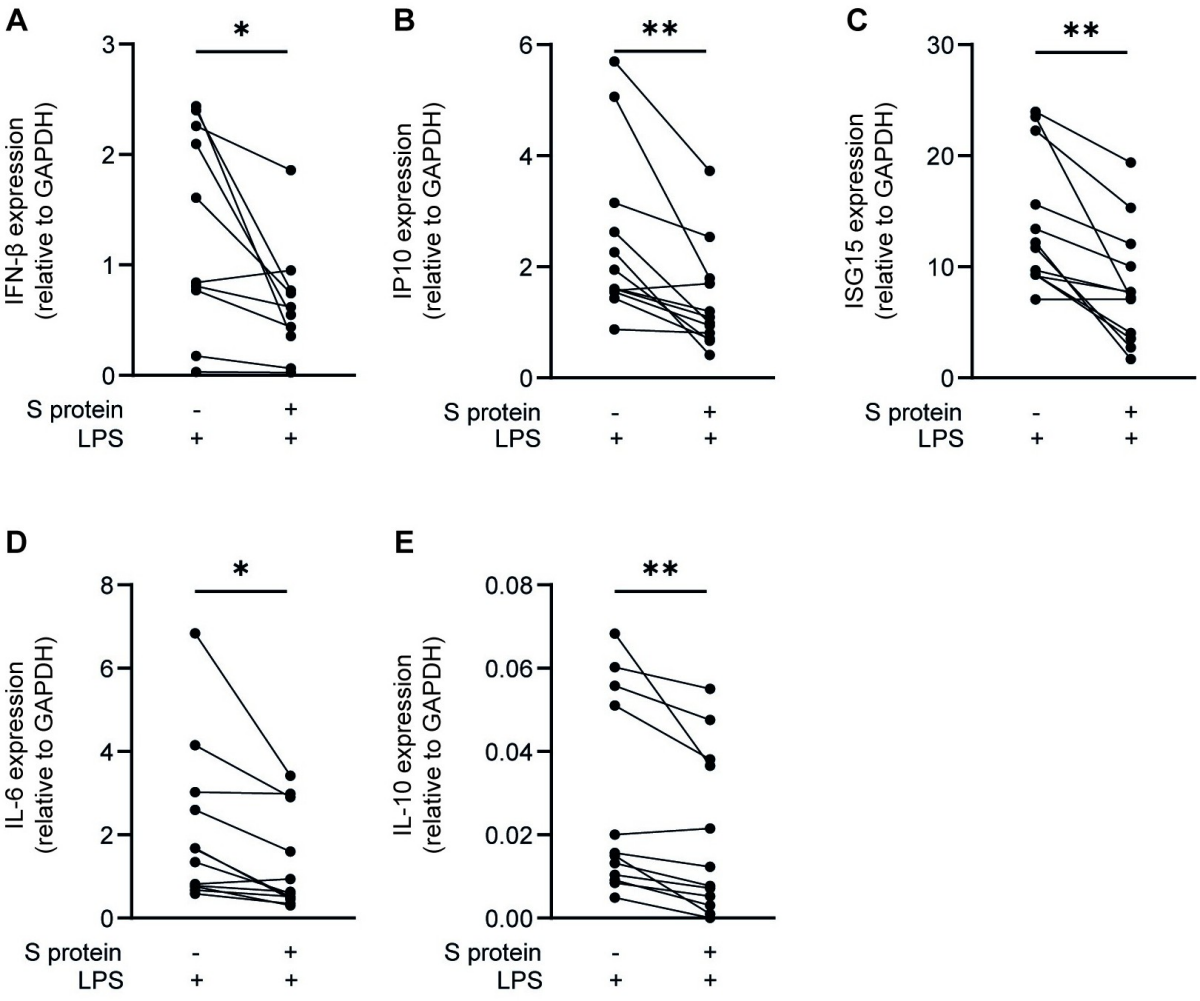
S protein modulates TLR4-mediated immune responses by DCs. (A-E) DCs were exposed to S protein prior to addition of TLR4 agonist LPS. After 2h or 6h incubation, cells were lysed and mRNA levels of IFN-β (A) were determined after 2h, and mRNA expression levels of IP10 (B), ISG15 (C) and cytokines IL-6 (D) and IL-10 (E) were determined after 6h by qPCR. Data show expression for n = 10 donors obtained in 5 separate experiments (2h stimulation) or n = 12 donors obtained in 6 separate experiments (6h stimulation). Statistical analysis was performed using student’s *t*-test. **p<0.01; *p<0.05.

We next determined the effect of SARS-CoV-2 on TLR4-induced immune responses. DCs from healthy donors were exposed to recombinant S protein before adding TLR4 agonist lipopolysaccharide (LPS) and type I IFN and cytokine responses were determined. LPS alone induced mRNA levels of IFN-β and ISGs IP10 and ISG15, and cytokines interleukin (IL)-6 and IL-10 (Fig 2A–2E). Notably, pre-exposure to recombinant S protein significantly reduced mRNA levels of IFN-β, IP10 and ISG15 as well as IL-6 and IL-10. As published before, S protein alone did not induce any type I IFN or cytokine responses [19]. Our data therefore strongly suggest that SARS-CoV-2 S protein suppresses both TLR4-induced type I IFN and cytokine responses.

### SARS-CoV-2 S protein does not directly affect TLR4 signaling

**Fig 3:**
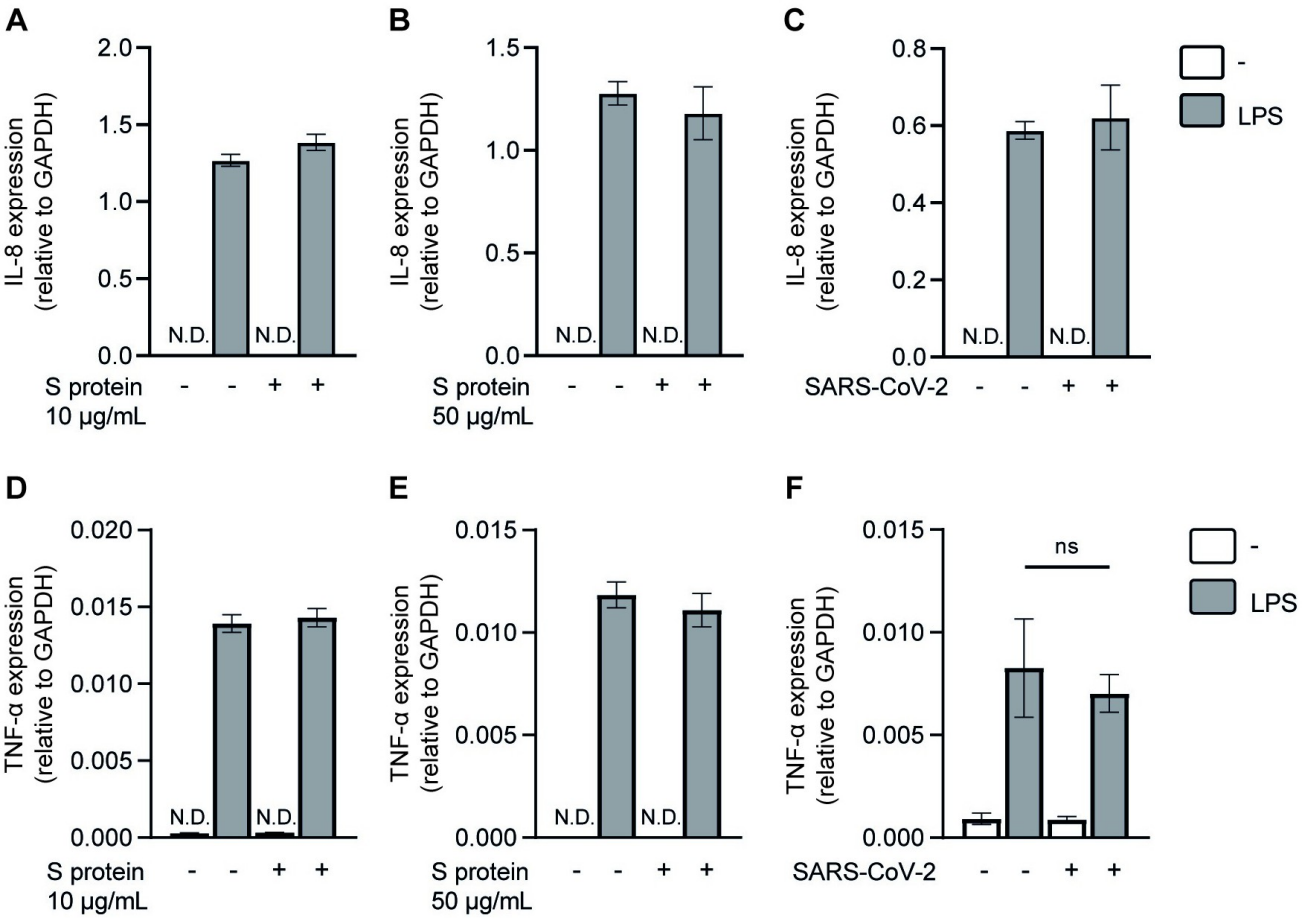
S protein or SARS-CoV-2 primary isolate does not sterically hinder binding of LPS to TLR4. (A-F) HEK293/TLR4 cells were pre-incubated for 2h with a low (A, D) or high (B, E) concentration of S protein or SARS-CoV-2 primary isolate (C, F) before exposure to TLR4 agonist LPS. After 24h incubation, cells were lysed and mRNA levels of IL-8 (A-C) and TNF-α (D-F) were determined by qPCR. Data show the mean values and SEM obtained in three separate experiments. Statistical analysis was performed using one-way ANOVA. ns = non-significant; N.D. = not detected.

SARS-CoV-2 has been suggested to interact with TLR4 [17,20,21] and therefore we investigated whether S protein could sterically hinder the binding of LPS using a TLR4-expressing HEK293 cell line (HEK293/TLR4). HEK293 cells do not inherently express TLRs or DNA sensors, but ectopic expression and triggering of TLR4 leads to the production of the cytokine IL-8. In contrast to parental HEK293 cells, incubation of HEK293/TLR4 cells with LPS induced IL-8 production (Fig 3A and 3B) [19]. Pre-incubation with a low and high concentration of recombinant S protein did not affect LPS-induced IL-8 expression by the HEK293/TLR4 cells (Fig 3A and 3B). Additionally, pre-incubation of HEK293/TLR4 cells with SARS-CoV-2 primary isolate (hCoV-19/Italy) prior to exposure to LPS did also not affect IL-8 production (Fig 3C). Whilst IL-8 is the primary readout in this assay, we aimed to confirm our findings by determining other cytokines. TLR4-mediated IFN responses were not detected, but the inflammatory cytokine TNF-α was induced upon TLR4 triggering, and remained unaffected by pre-incubation with S protein or SARS-CoV-2 primary isolate (Fig 3D–3F). Taken together, these results suggest that neither S protein nor SARS-CoV-2 primary isolate directly suppress TLR4 signaling either by sterically hindering binding of LPS to TLR4 or direct modulation of TLR4 signaling.

### SARS-CoV-2 S protein binds DC-SIGN expressed by DCs

**Fig 4:**
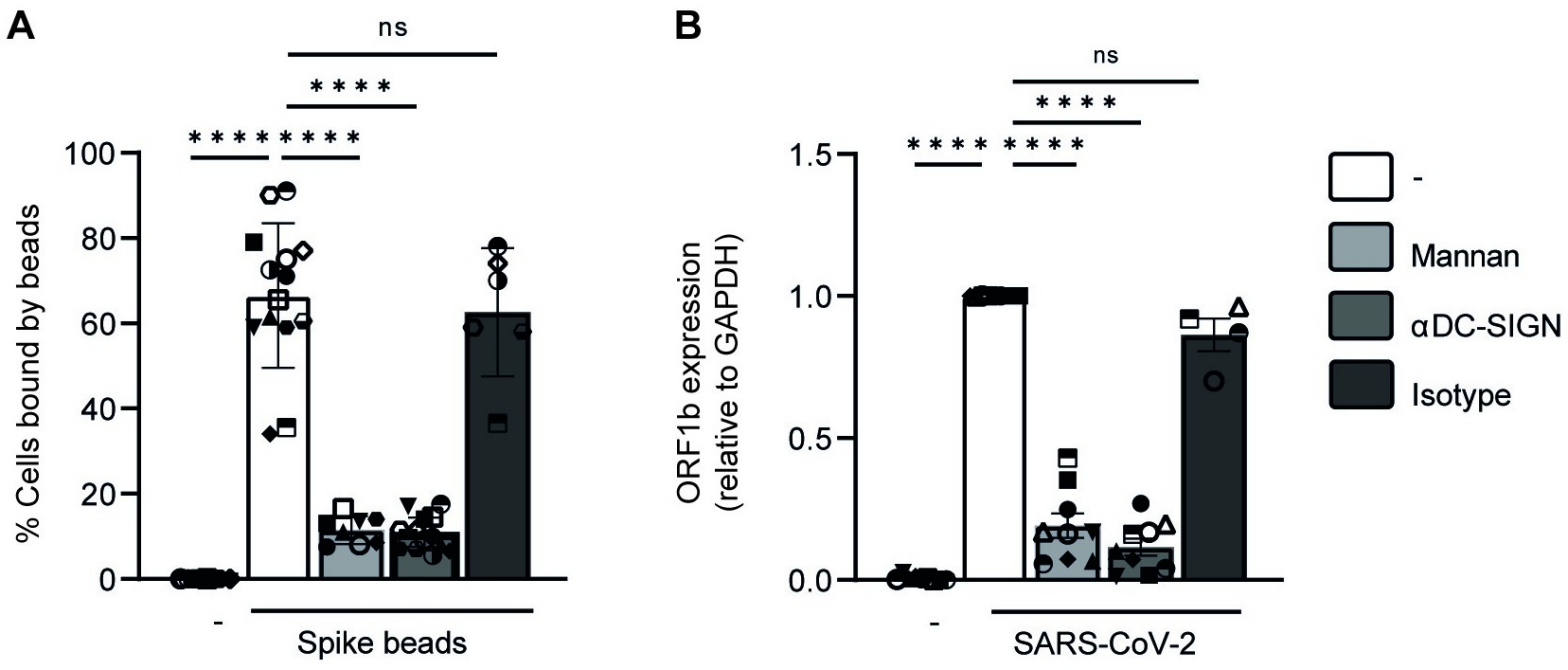
S protein binds DC-SIGN expressed by DCs. (A) DCs were exposed to S-protein-coated fluorescent beads in the absence or presence of CLR block mannan, DC-SIGN blocking antibodies, or an isotype control after which S protein binding to DCs was determined by flow cytometry. (B) DCs were exposed to SARS-CoV-2 primary isolate in the absence or presence of CLR block mannan, DC-SIGN blocking antibodies, or an isotype control after which virus binding to DCs was determined by qPCR. SARS-CoV-2 binding was set at 1 in cells treated with SARS-CoV-2 primary isolate. Data show the mean values and SEM. Statistical analysis was performed using one-way ANOVA. Data represent n = 8–14 donors obtained in 6 separate experiments performed in triplo (A) or n = 4–9 donors obtained in 5 separate experiments performed in triplo (B) with each symbol representing a different donor. ****p<0.0001; ns = non-significant.

Next we investigated whether crosstalk with the C-type lectin receptor (CLR) DC-SIGN is involved in the modulation of TLR4 signaling. Previous reports with different pathogens have shown that DC-SIGN signaling modulates immune responses by DCs [22–25] and SARS-CoV-2 S protein has been shown to interact with DC-SIGN [26,27]. However, whilst these reports show SARS-CoV-2 S protein binding to DC-SIGN-overexpressing cell lines, the direct binding of S protein to DC-SIGN expressed by primary DCs has not yet been elucidated. Therefore we investigated whether human DCs interact with SARS-CoV-2 S protein via DC-SIGN using a S-protein-labeled fluorescent bead binding assay [28]. Notably, S protein strongly bound to DCs and binding was abrogated by the CLR inhibitor mannan and blocking antibodies against DC-SIGN (Fig 4A). Moreover, DCs efficiently captured SARS-CoV-2 virus particles and binding was blocked by mannan as well as anti-DC-SIGN antibodies (Fig 4B). Isotype controls did neither affect the binding of S protein nor SARS-CoV-2 virus particles to DCs. These data strongly suggest that SARS-CoV-2 binds DC-SIGN on primary DCs via envelope glycoprotein S.

### SARS-CoV-2 suppresses TLR4-mediated DC activation via DC-SIGN

**Fig 5:**
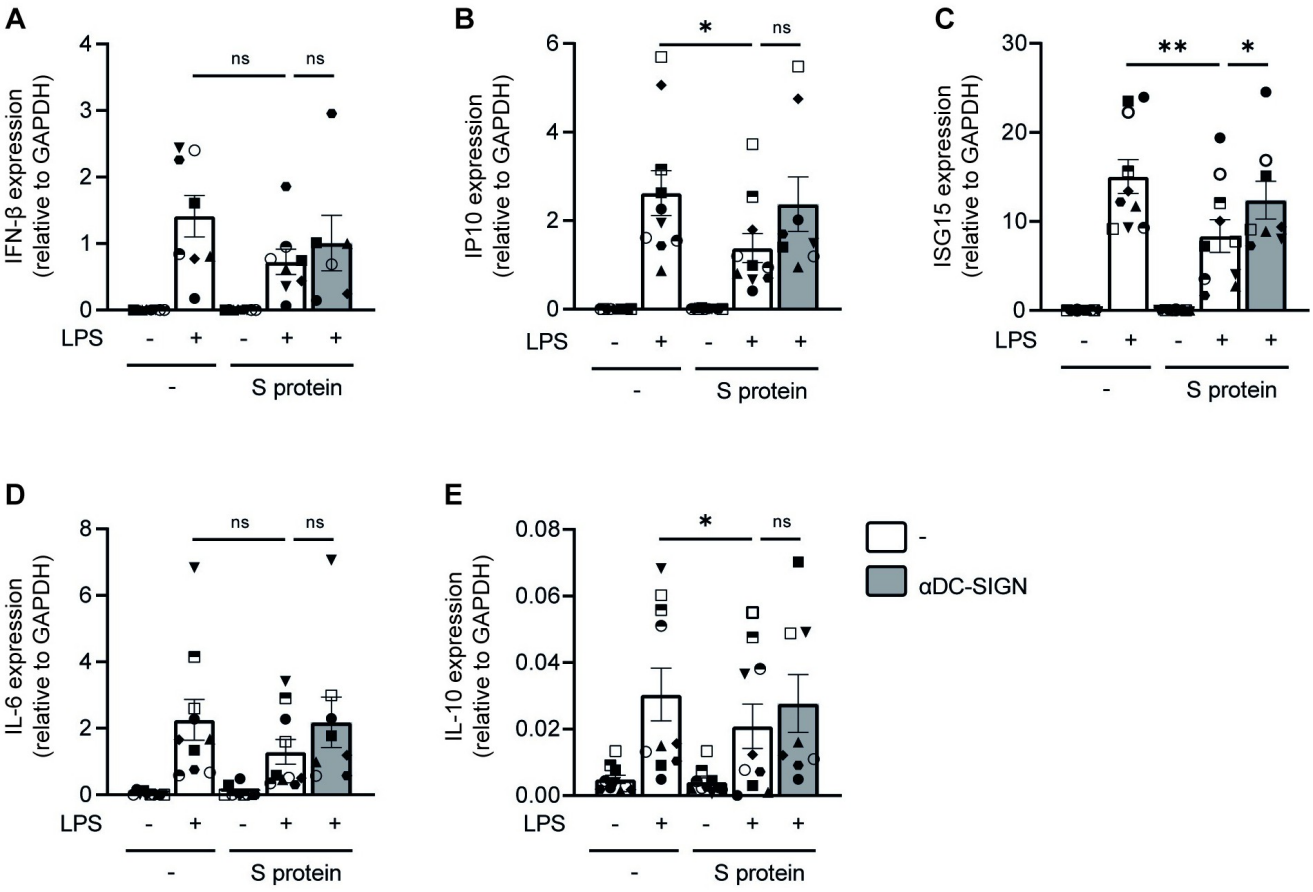
S protein suppresses TLR4-induced immunity via DC-SIGN. (A-B) DCs were incubated with S protein in the absence or presence of anti-DC-SIGN blocking antibodies before exposure to LPS. After 2h or 6h incubation, DCs were lysed and mRNA transcription of IFN-β (A), IP10 (B), ISG15 (C), IL-6 (D) and IL-10 (E) was determined by qPCR. Data show the mean values and SEM. Statistical analysis was performed using one-way ANOVA with Tukey’s multiple comparison’s test for mixed-effects analysis. Data represent n = 6–8 donors obtained in 4 separate experiments (2h stimulation) or n = 8–10 donors obtained in 6 separate experiments (6h stimulation) with each symbol representing a different donor. **p<0.01; *p<0.05; ns = non-significant.

Next we investigated whether SARS-CoV-2 suppresses TLR4 functionality via DC-SIGN. DCs from healthy donors were treated with recombinant SARS-CoV-2 S protein prior to LPS stimulation in presence or absence of antibodies against DC-SIGN, and type I IFN and cytokine responses were determined. Exposure to S protein decreased LPS-induced mRNA levels of IFN-β (p = ns), IP10 (p = 0.017) and ISG15 (p = 0.0059) (Fig 5A–5C). Antibodies against DC-SIGN restored IP10 (p = ns) and ISG15 (p = 0.025) expression to levels observed with LPS alone (Fig 5A–5C). IFN-β expression was not affected by antibodies against DC-SIGN. Similarly, exposure to S protein decreased LPS-induced mRNA levels of IL-6 (p = ns), and IL-10 (p = 0.042) (Fig 5D and 5E). S protein-mediated suppression of IL-6 and IL-10 was restored by blocking DC-SIGN, albeit not significantly and varied amongst DC donors (Fig 5D and 5E). These data suggest that DC-SIGN binding by S protein suppresses TLR4 signaling.

**Fig 6:**
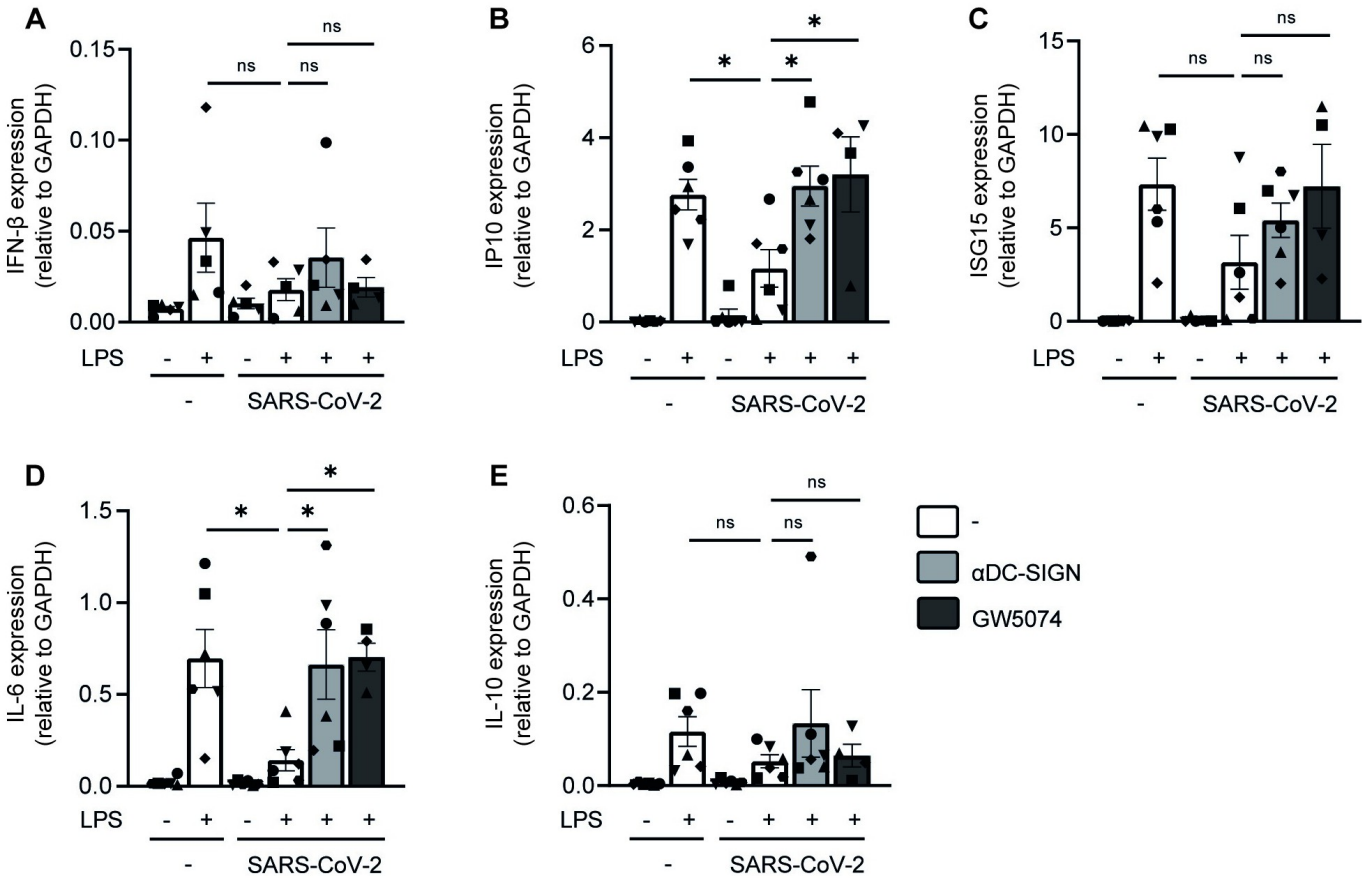
SARS-CoV-2 primary isolate suppresses DC immunity via DC-SIGN. (A-B) DCs were incubated with SARS-CoV-2 primary isolate in the absence or presence of anti-DC-SIGN blocking antibodies before exposure to LPS. After 2h or 6h incubation, DCs were lysed and mRNA transcription of IFN-β (A), IP10 (B), ISG15 (C), IL-6 (D) and IL-10 (E) was determined by qCPR. Data show the mean values and SEM of n = 4–5 donors obtained in 3 separate experiments (2h stimulation) or n = 4–6 donors obtained in 3 separate experiments (6h stimulation) with each symbol representing a different donor. Statistical analysis was performed using one-way ANOVA with Tukey’s multiple comparison’s test. *p<0.05; ns = non-significant.

Next we investigated whether SARS-CoV-2 primary isolate suppresses LPS-induced immune responses by DCs. Previously we have shown that SARS-CoV-2 primary isolate does not induce type I IFN and cytokine responses by DCs [19]. Strikingly, pre-incubation of DCs with SARS-CoV-2 primary isolate prior to exposure to LPS suppressed mRNA levels of IFN-β (p = ns), ISGs IP10 (p = 0.045) and ISG15 (p = ns), as well as cytokines IL-6 (p = 0.014) and IL-10 (p = ns) (Fig 6A–6E). Moreover, anti-DC-SIGN antibodies restored expression of type I IFN and cytokine responses to levels observed with LPS alone (Fig 6A–6E), although this effect was only significant for IP10 (p = 0.02) and IL-6 (p = 0.02). DC-SIGN signaling via mannose-expressing pathogens triggers Raf-1 activation leading to immune modulation [29]. We therefore studied whether a small molecule inhibitor of Raf-1 (GW5074) affects SARS-CoV-2-suppression of LPS signaling. Although Raf-1 inhibition did not affect SARS-CoV-2 suppression of IFN-β (p = ns), the expression of ISGs IP10 (p = 0.02) and ISG15 (p = ns) as well as cytokines IL-6 (p = 0.03) and IL-10 (p = ns) were restored by inhibiting Raf-1 to levels observed for LPS alone (Fig 6A–6E). The differences in significance are likely due to donor variability. These results suggest that SARS-CoV-2 modulates DC activation through DC-SIGN, thereby disabling DCs to respond to bacterial superinfections during COVID-19.

## Discussion

The SARS-CoV-2 pandemic has made an enormous impact all over the world. The high morbidity and mortality rates are not merely due to SARS-CoV-2 infection and aberrant immune responses against the virus, but are also due to superinfections [6–12]. Hospitalized patients with severe COVID-19 are susceptible to superinfections with other viruses, fungi or bacteria. Often these superinfections are caused by bacteria leading to pneumonia, bacteremia and sepsis [30,31]. Damage inflicted on lung tissue by SARS-CoV-2, and mechanical stress caused by intubations are major reasons for the spread of certain bacteria in the lungs and throughout the body [9,30,31]. However, other mechanisms might underlie increased susceptibility to bacterial superinfections, such as decreased function of DCs during COVID-19 [32]. Here we have identified a novel pathway activated by SARS-CoV-2 that suppresses TLR4, the major bacterial TLR on human DCs. Our data strongly suggest that SARS-CoV-2 interacts with the C-type lectin receptor DC-SIGN leading to Raf-1-mediated suppression of TLR4 signaling. We observed suppression of both type I IFN and cytokine responses and these inflammatory mediators are crucial in the defense against bacterial infections [33,34].

Bacterial superinfections are caused by both Gram-positive bacteria, including *Streptococcus pneumoniae* and Gram-negative bacteria including *P*. *aeruginosa* and *K*. *pneumoniae* [12]. Notably, DCs responded similarly to stimulation with Gram-positive stimuli Pam3CSK4 and LTA, irrespective of pre-exposure to recombinant SARS-CoV-2 S protein. Moreover, DCs in presence or absence of S protein also reacted similarly to the Gram-negative stimulus flagellin, whereas DCs were significantly less responsive towards LPS in the presence of S protein. These findings led us to investigate how TLR4 binding or signaling was affected. Another study reported decreased functioning of DCs from COVID-19 patients towards TLR triggers during acute SARS-CoV-2 infection as DC activation upon TLR3, TLR4, TLR7 and TLR8 triggering was suppressed [35]. Here we observed that SARS-CoV-2 specifically affected TLR4 signaling. As we have used DCs from healthy donors, our data suggest that SARS-CoV-2 as well as S protein directly affects TLR4 signaling.

Some pathogens are known to mimic pathogen or host structures to remain hidden from immune detection or become more pathogenic by inducing alternative signaling [36–40]. Previous research suggests that S protein binds TLR4 to trigger immune activation [17,20,21, 41]. We have shown that S protein does not activate DCs through TLR4 triggering [19]; however, S protein might still bind TLR4 and thereby inhibit TLR4 signaling. TLR4 activation in HEK293 cells ectopically expressing TLR4 was neither affected by recombinant SARS-CoV-2 S protein nor SARS-CoV-2 infectious virus. These data strongly suggest that SARS-CoV-2 does neither sterically block binding of LPS to TLR4 nor directly inhibit TLR4 signaling. Besides TLRs, DCs express many different PRRs involved in virus binding. An important PRR family are the CLRs that interact with pathogens via carbohydrates and have been shown to induce signaling that directs or modulates immune responses [42,43]. In particular, the CLR DC-SIGN is expressed by DCs and macrophages, and recognizes high-mannose-containing glycoproteins on the surface of pathogens [44,45]. Previous research has shown that DC-SIGN modulates immune activation towards various pathogens [25,45,46]. ManLAM, a highly mannosylated cell-wall component of *Mycobacterium (M*.*) tuberculosis*, interacts with DC-SIGN resulting in an altered immune response through crosstalk between DC-SIGN and TLR4 [22,25]. Interaction between ManLAM and DC-SIGN modulates TLR4 signaling via Raf-1 resulting in phosphorylation and acetylation of NFκB, which enhances induction of IL-10, IL-12 and IL-6 [22,25]. Similarly, envelope glycoprotein of HIV-1 enhances LPS-induced IL-10, IL-12 and IL-6 responses via DC-SIGN [29]. However, our data strongly suggest that SARS-CoV-2 interaction with DC-SIGN suppresses TLR4 signaling via Raf-1. Thus, DC-SIGN modulation of immunity is strongly dependent on the PRR triggered as well as the pathogen that is recognized by DC-SIGN, which might thereby underlie the observed differences between SARS-CoV-2 and HIV-1. It is currently unclear what causes the different effects on TLR4 signaling induced by HIV-1 or SARS-CoV-2 on DCs. DC-SIGN triggering strongly depends on the sugar moieties expressed on the viral envelope [46]. Whilst both HIV-1 and SARS-CoV-2 express highly mannosylated envelope proteins, the HIV-1 envelope glycoprotein gp120 is mostly composed of oligomannose structures [47], whereas the SARS-CoV-2 envelope S glycoprotein mainly includes complex glycosylation [48,49]. In addition, glycoprotein density between HIV-1 and SARS-CoV-2 virions might account for differences as density of glycoproteins on virions might lead to different receptor crosslinking and signaling [50,51]. These differences in the envelope glycoproteins might explain the different downstream effects of DC-SIGN signaling. Moreover, DCs can be infected by HIV-1, whilst DCs are not infected by SARS-CoV-2. Possibly internalization of HIV-1 in the DC’s endosomal compartment induces complementary intracellular signaling that is not induced by SARS-CoV-2. However, for SARS-CoV-2 we can also not exclude involvement of other receptors. Further research on the effect of DC-SIGN triggering during SARS-CoV-2 infection is required to elucidate underlying mechanisms. In addition, it is important to note that also coinfections or superinfections with other viruses and fungi were reported [6,12,52–54]. It would be interesting to further investigate how superinfections with different bacteria, viruses or fungi might affect DC function during COVID-19. In conclusion, our data suggest that SARS-CoV-2 S protein-mediated DC-SIGN crosstalk affects TLR4-induced immunity, which might underlie bacterial superinfections during COVID-19.

## Materials and methods

### Ethics approval statement

This study was performed in accordance with the ethical principles set out in the declaration of Helsinki and was approved by the institutional review board of the Amsterdam University Medical Centers, location AMC Medical Ethics Committee and the Ethics Advisory Body of Sanquin Blood Supply Foundation (Amsterdam, Netherlands). Written informed consent was obtained from all participants.

### Cell lines

The Simian kidney cell line VeroE6 (ATCC CRL-1586) was maintained in CO_2_-independent medium (Gibco Life Technologies, Gaithersburg, Md.) supplemented with 10% fetal calf serum (FCS), 2mM L-glutamine and penicillin/streptomycin (Invitrogen). Cultures were maintained at 37°C without CO_2_. The human embryonic kidney (HEK) 293 cells (ATCC CRL-11268) were maintained in Iscove’s modified Dulbecco’s medium (IMDM) (Gibco Life Technologies) containing 10% FCS, L-glutamine, and 1% penicillin/streptomycin. Cultures were maintained at 37°C and 5% CO_2_. HEK293 cells stably transfected with TLR4 cDNA (HEK/TLR4) were a kind gift from Dr. T. Golenbock [15]. Cells were split and seeded into flat-bottom 96-well plates (Corning) and left to attach for 24h, before performing further experiments.

### Primary cells

This study was performed in accordance with the ethical principles set out in the declaration of Helsinki and was approved by the institutional review board of the Amsterdam University Medical Centers, location AMC Medical Ethics Committee and the Ethics Advisory Body of Sanquin Blood Supply Foundation (Amsterdam, Netherlands). Written informed consent was obtained from all participants. Human CD14^+^ monocytes were isolated from the blood of healthy volunteer donors and subsequently differentiated into monocyte-derived dendritic cells (DCs) as described before [24]. In short, the isolation of monocytes from buffy coats was performed by density centrifugation on Lymphoprep (Nycomed) and Percoll (Pharmacia). Monocytes were cultured in Roswell Park Memorial Institute (RPMI) 1640 (Gibco), supplemented with 10% FCS, 2mM L-glutamin (Invitrogen), and 1% penicillin/streptomycin. Differentiation into DCs was performed by the addition of cytokines IL-4 (500U/mL) and GM-CSF (800U/mL) (both Gibco). After 4 days of differentiation, DCs were seeded at 1 x 10^6^/mL in a round-bottom 96-well plate (Corning). After 2 days of recovery, DCs were stimulated as described below.

### SARS-CoV-2 (hCoV-19/Italy) virus production

The following reagent was obtained from Dr. Maria R. Capobianchi through BEI Resources, NIAID, NIH:SARS-related coronavirus 2, Isolate Italy-INMI1, NR-52284, originally isolated in January 2020 in Rome, Italy. SARS-CoV-2 virus productions were performed as described before [19,55] In brief, VeroE6 cells were inoculated with the SARS-CoV-2 primary isolate and incubated for 48h, after which virus supernatant was harvested. Tissue culture infectious dose (TCID50) was determined on VeroE6 cells by MTT assay. MTT staining is indicative of cell viability and can be measured using a spectrometer. The virus titer was determined as TCID50/mL and calculated based on the Reed Muench method [56] as described before [55].

### Reagents and stimulations

DCs were left unstimulated or exposed to 10 μg/mL SARS-CoV-2 S protein (Bio-techne) for 1h, after which DCs were exposed to the following TLR stimuli: 10μg/mL Pam3CSK4 (Invivogen), 10 μg/mL Poly(I:C) (Invivogen), 10 ng/mL LPS from *Salmonella typhi* (Sigma), 10 μg/mL flagellin from *Bacillus subtilis* (Invivogen), 10 μg/mL lipotechoic acid from *Staphylococcus aureus* (Invivogen). To investigate the contribution of DC-SIGN and Raf-1, cells were pre-incubated with 20μg/mL anti-DC-SIGN blocking antibody AZN-D1 for 30 min, or with 1μM GW5074 (Calbiochem) for 2h, respectively, before adding S protein. For exposure to SARS-CoV-2 (hCoV-19/Italy), DCs were incubated with inhibitors prior to exposure to SARS-CoV-2 TCID1000 for 1h, and then to LPS for 2 or 6h, after which cells were lysed.

Similarly, HEK293 and HEK/TLR4 cells were incubated for 2h with SARS-CoV-2 S protein or SARS-CoV-2 (hCoV-19/Italy), after which LPS was added. Cells were lysed after 24h for qPCR analysis.

### RNA isolation and quantitative real-time PCR

Cells exposed to SARS-CoV-2 primary isolate (hCoV-19/Italy) were lysed and RNA was isolated with the QIAamp Viral RNA Mini Kit (Qiagen) according to the manufacturer’s protocol. cDNA was synthesized using the M-MLV reverse-transcriptase kit (Promega). Before further application, cDNA was diluted 1 in 5 in depc. Cells exposed to SARS-CoV-2 S protein were lysed and RNA was isolated with the RNA Catcher PLUS kit (Invivogen) according to the manufacturer’s instructions. Subsequently, cDNA was synthesized with a reverse-transcriptase kit (Promega). PCR amplification was performed in the presence of SYBR green (Thermofisher) in a 7500 Fast Realtime PCR system (ABI). Specific primers were designed using Primer Express 2.0 (Applied Biosystems). The comparative delta Ct method was used to normalize the amount of target mRNA to the expression of household gene GAPDH.

The following primers were used:

GAPDH: F_CCATGTTCGTCATGGGTGTG; R_GGTGCTAAGCAGTTGGTGGTG; IFNB: F_ACAGACTTACAGGTTACCTCCGAAAC; R_CATCTGCTGGTTGAAGAATGCTT; ISG15: F_ TTTGCCAGTACAGGAGCTTGTG; R_ GGGTGATCTGCGCCTTCA; CXCL10: F_ CGCTGTACCTGCATCAGCAT; R_ CATCTCTTCTCACCCTTCTTTTTCA; IL-6: F_TGCAATAACCACCCCTGACC; R_TGCGCAGAATGAGATGAGTTG; IL-10: F_GAGGCTACGGCGCTGTCAT; R_CCACGGCCTTGCTCTTGTT; ORF1b: F_TGGGGTTTTACAGGTAACCT; R_AACACGCTTAACAAAGCACT; TNF: F_ CCAAGCCCTGGTATGAGCC; R_ GCCGATTGATCTCAGCGC.

### Bead binding and SARS-CoV-2 isolate (hCoV-19/Italy) binding assays

To investigate ligand-receptor interactions, we used a fluorescent bead binding assay as described before [28]. PerCP fluorescent streptavidin beads were coated with biotinylated S protein (Bio-techne). DCs were seeded at a density of 50.000 cell/well in a 96-well V-bottom plate in TSA (TSA: 0.5% bovine serum albumin (BSA) in TSM (200mM Tris, 1500mM NaCl, 10mM CaCl_2_, 20mM MgCl_2_) pH 7.4). Subsequently, cells were incubated with TSA, 20μg/mL anti-DC-SIGN antibody AZN-D1, 100μg/mL mannan, or 20μg/mL anti-langerin antibody isotype 10E2 for 30 min at 37°C. Beads were added to each corresponding well in a 1:20 dilution and incubated at 37°C for 45 min. After washing once with TSA, cells were resuspended in TSA and adhesion was measured on a FACS Canto flow cytometer (BD Biosciences). The data was analyzed using FlowJo V10 software (Treestar).

To assess virus binding, DCs were exposed to 20μg/mL anti-DC-SIGN blocking antibody AZN-D1, 50μg/mL mannan, or 20μg/mL anti-langerin antibody isotype 10E2 for 30 min at 37°C prior to incubation with SARS-CoV-2 isolate (hCoV-19/Italy) for 2h at 4°C. After 2h, cells were washed extensively with phosphate-buffered saline (PBS) to remove unbound virus and subsequently lysed with AVL buffer (Qiagen). RNA and cDNA were prepared as described above, and the amount of virus bound was determined with qPCR using ORF1b primers [57].

### Statistics

Graphpad Prism version 8 (GraphPad Software) was used to generate all graphs and to perform statistical analyses. For pairwise comparisons, a Student’s *t*-test was used. Multiple comparisons within groups were performed using a one-way ANOVA with a Tukey’s multiple comparisons test, or two-way ANOVA with a Tukey’s multiple comparisons test, where indicated. p<0.05 were considered statistically significant.

## Supporting information

S1 Data Excel spreadsheet containing the underlying numerical data for Figs 1–6 in separate sheets. (XLSX)

## References

[1] Harrison AG, Lin T, Wang P. Mechanisms of SARS-CoV-2 Transmission and Pathogenesis. Trends Immunol. 2020;41(12):1100–15. doi: 10.1016/j.it.2020.10.004 33132005 PMC7556779

[2] Wang D, Hu B, Hu C, Zhu F, Liu X, Zhang J, et al. Clinical Characteristics of 138 Hospitalized Patients With 2019 Novel Coronavirus-Infected Pneumonia in Wuhan, China. JAMA. 2020;323(11):1061–9. doi: 10.1001/jama.2020.1585 32031570 PMC7042881

[3] Wiersinga WJ, Rhodes A, Cheng AC, Peacock SJ, Prescott HC. Pathophysiology, Transmission, Diagnosis, and Treatment of Coronavirus Disease 2019 (COVID-19): A Review. JAMA. 2020;324(8):782–93. doi: 10.1001/jama.2020.12839 32648899

[4] Hoffmann M, Kleine-Weber H, Schroeder S, Kruger N, Herrler T, Erichsen S, et al. SARS-CoV-2 Cell Entry Depends on ACE2 and TMPRSS2 and Is Blocked by a Clinically Proven Protease Inhibitor. Cell. 2020;181(2):271–80 e8. doi: 10.1016/j.cell.2020.02.052 32142651 PMC7102627

[5] Letko M, Marzi A, Munster V. Functional assessment of cell entry and receptor usage for SARS-CoV-2 and other lineage B betacoronaviruses. Nat Microbiol. 2020;5(4):562–9. doi: 10.1038/s41564-020-0688-y 32094589 PMC7095430

[6] Garcia-Vidal C, Sanjuan G, Moreno-Garcia E, Puerta-Alcalde P, Garcia-Pouton N, Chumbita M, et al. Incidence of co-infections and superinfections in hospitalized patients with COVID-19: a retrospective cohort study. Clin Microbiol Infect. 2021;27(1):83–8. doi: 10.1016/j.cmi.2020.07.041 32745596 PMC7836762

[7] Langford BJ, So M, Raybardhan S, Leung V, Westwood D, MacFadden DR, et al. Bacterial co-infection and secondary infection in patients with COVID-19: a living rapid review and meta-analysis. Clin Microbiol Infect. 2020;26(12):1622–9. doi: 10.1016/j.cmi.2020.07.016 32711058 PMC7832079

[8] Omoush SA, Alzyoud JAM. The Prevalence and Impact of Coinfection and Superinfection on the Severity and Outcome of COVID-19 Infection: An Updated Literature Review. Pathogens. 2022;11(4). doi: 10.3390/pathogens11040445 35456120 PMC9027948

[9] Pickens CO, Gao CA, Cuttica MJ, Smith SB, Pesce LL, Grant RA, et al. Bacterial Superinfection Pneumonia in Patients Mechanically Ventilated for COVID-19 Pneumonia. Am J Respir Crit Care Med. 2021;204(8):921–32. doi: 10.1164/rccm.202106-1354OC 34409924 PMC8534629

[10] Catano-Correa JC, Cardona-Arias JA, Porras Mancilla JP, Garcia MT. Bacterial superinfection in adults with COVID-19 hospitalized in two clinics in Medellin-Colombia, 2020. PLoS One. 2021;16(7):e0254671. 34255801 10.1371/journal.pone.0254671 PMC8277025

[11] Feldman C, Anderson R. The role of co-infections and secondary infections in patients with COVID-19. Pneumonia (Nathan). 2021;13(1):5. doi: 10.1186/s41479-021-00083-w 33894790 PMC8068564

[12] Musuuza JS, Watson L, Parmasad V, Putman-Buehler N, Christensen L, Safdar N. Prevalence and outcomes of co-infection and superinfection with SARS-CoV-2 and other pathogens: A systematic review and meta-analysis. PLoS One. 2021;16(5):e0251170. doi: 10.1371/journal.pone.0251170 33956882 PMC8101968

[13] Janeway CA Jr., Medzhitov R. Innate immune recognition. Annu Rev Immunol. 2002;20:197–216. doi: 10.1146/annurev.immunol.20.083001.084359 11861602

[14] Medzhitov R. Toll-like receptors and innate immunity. Nat Rev Immunol. 2001;1(2):135–45. doi: 10.1038/35100529 11905821

[15] Chow JC, Young DW, Golenbock DT, Christ WJ, Gusovsky F. Toll-like receptor-4 mediates lipopolysaccharide-induced signal transduction. J Biol Chem. 1999;274(16):10689–92. doi: 10.1074/jbc.274.16.10689 10196138

[16] Shirato K, Kizaki T. SARS-CoV-2 spike protein S1 subunit induces pro-inflammatory responses via toll-like receptor 4 signaling in murine and human macrophages. Heliyon. 2021;7(2):e06187. doi: 10.1016/j.heliyon.2021.e06187 33644468 PMC7887388

[17] Zhao Y, Kuang M, Li J, Zhu L, Jia Z, Guo X, et al. SARS-CoV-2 spike protein interacts with and activates TLR41. Cell Res. 2021;31(7):818–20. doi: 10.1038/s41422-021-00495-9 33742149 PMC7975240

[18] Petruk G, Puthia M, Petrlova J, Samsudin F, Stromdahl AC, Cerps S, et al. SARS-CoV-2 spike protein binds to bacterial lipopolysaccharide and boosts proinflammatory activity. J Mol Cell Biol. 2020;12(12):916–32. doi: 10.1093/jmcb/mjaa067 33295606 PMC7799037

[19] van der Donk LEH, Eder J, van Hamme JL, Brouwer PJM, Brinkkemper M, van Nuenen AC, et al. SARS-CoV-2 infection activates dendritic cells via cytosolic receptors rather than extracellular TLRs. Eur J Immunol. 2022;52(4):646–55. doi: 10.1002/eji.202149656 35099061 PMC9015339

[20] Aboudounya MM, Heads RJ. COVID-19 and Toll-Like Receptor 4 (TLR4): SARS-CoV-2 May Bind and Activate TLR4 to Increase ACE2 Expression, Facilitating Entry and Causing Hyperinflammation. Mediators Inflamm. 2021;2021:8874339. doi: 10.1155/2021/8874339 33505220 PMC7811571

[21] Choudhury A, Mukherjee S. In silico studies on the comparative characterization of the interactions of SARS-CoV-2 spike glycoprotein with ACE-2 receptor homologs and human TLRs. J Med Virol. 2020;92(10):2105–13. doi: 10.1002/jmv.25987 32383269 PMC7267663

[22] Geijtenbeek TB, Van Vliet SJ, Koppel EA, Sanchez-Hernandez M, Vandenbroucke-Grauls CM, Appelmelk B, Van Kooyk Y. Mycobacteria target DC-SIGN to suppress dendritic cell function. J Exp Med. 2003;197(1):7–17. doi: 10.1084/jem.20021229 12515809 PMC2193797

[23] Gringhuis SI, Hertoghs N, Kaptein TM, Zijlstra-Willems EM, Sarrami-Forooshani R, Sprokholt JK, et al. HIV-1 blocks the signaling adaptor MAVS to evade antiviral host defense after sensing of abortive HIV-1 RNA by the host helicase DDX3. Nat Immunol. 2017;18(2):225–35. doi: 10.1038/ni.3647 28024153

[24] Mesman AW, Zijlstra-Willems EM, Kaptein TM, de Swart RL, Davis ME, Ludlow M, et al. Measles virus suppresses RIG-I-like receptor activation in dendritic cells via DC-SIGN-mediated inhibition of PP1 phosphatases. Cell Host Microbe. 2014;16(1):31–42. doi: 10.1016/j.chom.2014.06.008 25011106 PMC4159752

[25] Gringhuis SI, den Dunnen J, Litjens M, van Het Hof B, van Kooyk Y, Geijtenbeek TB. C-type lectin DC-SIGN modulates Toll-like receptor signaling via Raf-1 kinase-dependent acetylation of transcription factor NF-kappaB. Immunity. 2007;26(5):605–16. doi: 10.1016/j.immuni.2007.03.012 17462920

[26] Amraei R, Yin W, Napoleon MA, Suder EL, Berrigan J, Zhao Q, et al. CD209L/L-SIGN and CD209/DC-SIGN Act as Receptors for SARS-CoV-2. ACS Cent Sci. 2021;7(7):1156–65. doi: 10.1021/acscentsci.0c01537 34341769 PMC8265543

[27] Thepaut M, Luczkowiak J, Vives C, Labiod N, Bally I, Lasala F, et al. DC/L-SIGN recognition of spike glycoprotein promotes SARS-CoV-2 trans-infection and can be inhibited by a glycomimetic antagonist. PLoS Pathog. 2021;17(5):e1009576. doi: 10.1371/journal.ppat.1009576 34015061 PMC8136665

[28] Sprokholt JK, Hertoghs N, Geijtenbeek TB. Flow Cytometry-Based Bead-Binding Assay for Measuring Receptor Ligand Specificity. Methods Mol Biol. 2016;1390:121–9. doi: 10.1007/978-1-4939-3335-8_8 26803626

[29] Gringhuis SI, den Dunnen J, Litjens M, van der Vlist M, Geijtenbeek TB. Carbohydrate-specific signaling through the DC-SIGN signalosome tailors immunity to Mycobacterium tuberculosis, HIV-1 and Helicobacter pylori. Nat Immunol. 2009;10(10):1081–8. doi: 10.1038/ni.1778 19718030

[30] Maslove DM, Sibley S, Boyd JG, Goligher EC, Munshi L, Bogoch II, Rochwerg B. Complications of Critical COVID-19: Diagnostic and Therapeutic Considerations for the Mechanically Ventilated Patient. Chest. 2022;161(4):989–98. doi: 10.1016/j.chest.2021.10.011 34655568 PMC8511547

[31] Wicky PH, Niedermann MS, Timsit JF. Ventilator-associated pneumonia in the era of COVID-19 pandemic: How common and what is the impact? Crit Care. 2021;25(1):153. doi: 10.1186/s13054-021-03571-z 33882991 PMC8059113

[32] Winheim E, Rinke L, Lutz K, Reischer A, Leutbecher A, Wolfram L, et al. Impaired function and delayed regeneration of dendritic cells in COVID-19. PLoS Pathog. 2021;17(10):e1009742. doi: 10.1371/journal.ppat.1009742 34614036 PMC8523079

[33] Boxx GM, Cheng G. The Roles of Type I Interferon in Bacterial Infection. Cell Host Microbe. 2016;19(6):760–9. doi: 10.1016/j.chom.2016.05.016 27281568 PMC5847370

[34] Giamarellos-Bourboulis EJ, Raftogiannis M. The immune response to severe bacterial infections: consequences for therapy. Expert Rev Anti Infect Ther. 2012;10(3):369–80. doi: 10.1586/eri.12.2 22397569

[35] Zhou R, To KK, Wong YC, Liu L, Zhou B, Li X, et al. Acute SARS-CoV-2 Infection Impairs Dendritic Cell and T Cell Responses. Immunity. 2020;53(4):864–77 e5. doi: 10.1016/j.immuni.2020.07.026 32791036 PMC7402670

[36] Gowthaman U, Eswarakumar VP. Molecular mimicry: good artists copy, great artists steal. Virulence. 2013;4(6):433–4. doi: 10.4161/viru.25780 23863600 PMC5359722

[37] Rahman MM, McFadden G. Modulation of NF-kappaB signalling by microbial pathogens. Nat Rev Microbiol. 2011;9(4):291–306. 21383764 10.1038/nrmicro2539 PMC3611960

[38] Rojas JM, Alejo A, Martin V, Sevilla N. Viral pathogen-induced mechanisms to antagonize mammalian interferon (IFN) signaling pathway. Cell Mol Life Sci. 2021;78(4):1423–44. doi: 10.1007/s00018-020-03671-z 33084946 PMC7576986

[39] Chathuranga K, Weerawardhana A, Dodantenna N, Lee JS. Regulation of antiviral innate immune signaling and viral evasion following viral genome sensing. Exp Mol Med. 2021;53(11):1647–68. doi: 10.1038/s12276-021-00691-y 34782737 PMC8592830

[40] Reddick LE, Alto NM. Bacteria fighting back: how pathogens target and subvert the host innate immune system. Mol Cell. 2014;54(2):321–8. doi: 10.1016/j.molcel.2014.03.010 24766896 PMC4023866

[41] Aboudounya MM, Holt MR, Heads RJ. SARS-CoV-2 Spike S1 glycoprotein is a TLR4 agonist, upregulates ACE2 expression and induces pro-inflammatory M1 macrophage polarisation. bioRxiv. 2021:2021.08.11.455921.

[42] Li D, Wu M. Pattern recognition receptors in health and diseases. Signal Transduct Target Ther. 2021;6(1):291. doi: 10.1038/s41392-021-00687-0 34344870 PMC8333067

[43] Geijtenbeek TB, Gringhuis SI. Signalling through C-type lectin receptors: shaping immune responses. Nat Rev Immunol. 2009;9(7):465–79. doi: 10.1038/nri2569 19521399 PMC7097056

[44] Geijtenbeek TB, Torensma R, van Vliet SJ, van Duijnhoven GC, Adema GJ, van Kooyk Y, Figdor CG. Identification of DC-SIGN, a novel dendritic cell-specific ICAM-3 receptor that supports primary immune responses. Cell. 2000;100(5):575–85. doi: 10.1016/s0092-8674(00)80693-5 10721994

[45] van Kooyk Y, Geijtenbeek TB. DC-SIGN: escape mechanism for pathogens. Nat Rev Immunol. 2003;3(9):697–709. doi: 10.1038/nri1182 12949494

[46] Gringhuis SI, Kaptein TM, Wevers BA, Mesman AW, Geijtenbeek TB. Fucose-specific DC-SIGN signalling directs T helper cell type-2 responses via IKKepsilon- and CYLD-dependent Bcl3 activation. Nat Commun. 2014;5:3898. 24867235 10.1038/ncomms4898

[47] Cao L, Diedrich JK, Kulp DW, Pauthner M, He L, Park SR, et al. Global site-specific N-glycosylation analysis of HIV envelope glycoprotein. Nat Commun. 2017;8:14954. doi: 10.1038/ncomms14954 28348411 PMC5379070

[48] Gong Y, Qin S, Dai L, Tian Z. The glycosylation in SARS-CoV-2 and its receptor ACE2. Signal Transduct Target Ther. 2021;6(1):396. doi: 10.1038/s41392-021-00809-8 34782609 PMC8591162

[49] Watanabe Y, Allen JD, Wrapp D, McLellan JS, Crispin M. Site-specific glycan analysis of the SARS-CoV-2 spike. Science. 2020;369(6501):330–3. doi: 10.1126/science.abb9983 32366695 PMC7199903

[50] Cummins NW, Rizza SA, Badley AD. How much gp120 is there? J Infect Dis. 2010;201(8):1273–4; author reply 4–5. 10.1086/651434 20225961

[51] Ke Z, Oton J, Qu K, Cortese M, Zila V, McKeane L, et al. Structures and distributions of SARS-CoV-2 spike proteins on intact virions. Nature. 2020;588(7838):498–502. doi: 10.1038/s41586-020-2665-2 32805734 PMC7116492

[52] Nowak MD, Sordillo EM, Gitman MR, Paniz Mondolfi AE. Coinfection in SARS-CoV-2 infected patients: Where are influenza virus and rhinovirus/enterovirus? J Med Virol. 2020;92(10):1699–700. doi: 10.1002/jmv.25953 32352574 PMC7267652

[53] Chen N, Zhou M, Dong X, Qu J, Gong F, Han Y, et al. Epidemiological and clinical characteristics of 99 cases of 2019 novel coronavirus pneumonia in Wuhan, China: a descriptive study. Lancet. 2020;395(10223):507–13. doi: 10.1016/S0140-6736(20)30211-7 32007143 PMC7135076

[54] Rawson TM, Moore LSP, Zhu N, Ranganathan N, Skolimowska K, Gilchrist M, et al. Bacterial and Fungal Coinfection in Individuals With Coronavirus: A Rapid Review To Support COVID-19 Antimicrobial Prescribing. Clin Infect Dis. 2020;71(9):2459–68. doi: 10.1093/cid/ciaa530 32358954 PMC7197596

[55] Bermejo-Jambrina M, Eder J, Kaptein TM, van Hamme JL, Helgers LC, Vlaming KE, et al. Infection and transmission of SARS-CoV-2 depend on heparan sulfate proteoglycans. EMBO J. 2021;40(20):e106765. doi: 10.15252/embj.2020106765 34510494 PMC8521309

[56] Reed LJ, Muench H. A simple method of estimating fifty per cent endpoints. American journal of epidemiology. 1938;27(3):493–7.

[57] Chu DKW, Pan Y, Cheng SMS, Hui KPY, Krishnan P, Liu Y, et al. Molecular Diagnosis of a Novel Coronavirus (2019-nCoV) Causing an Outbreak of Pneumonia. Clin Chem. 2020;66(4):549–55. doi: 10.1093/clinchem/hvaa029 32031583 PMC7108203

