# Supplementary material for: SARS-CoV-2 suppresses TLR4-induced immunity by dendritic cells via C-type lectin receptor DC-SIGN
Source: PLoS Pathog. 2023 Oct 16;19(10):e1011735. doi: 10.1371/journal.ppat.1011735 (PMC10602378; doi:10.1371/journal.ppat.1011735)
Supplement: Attachment — Submitted filename: Response to the reviewers_20230925.docx [file ppat.1011735.s002.docx]

**Response to the reviewers**

**Part I - Summary**
Please use this section to discuss strengths/weaknesses of study, novelty/significance, general execution and scholarship.

Reviewer #1: In this study, van de Donk and colleagues obtained several lines of evidence that strongly suggest that SARS-CoV-2 suppresses TLR4, the major bacterial TLR on human DCs and that SARS-CoV-2 interacts with DC-SIGN leading to Raf-1-mediated suppression of TLR4 signaling crucial in the defense against bacterial infections.
The paper is well written, data well oresented, and most importantly interesting.

Reviewer #2: In this manuscript, van der Donk et al. investigate dendritic cell (DC) function after SARS-CoV-2 exposure, following up on their earlier study (van der Donk et al. (2022) Eur. J. Immunol.) showing that SARS-CoV-2 does not activate extracellular TLRs or induce DC maturation. Here, they first show that dendritic cell exposure to SARS-CoV-2 spike protein dampens responsiveness to lipopolysaccharide (LPS), a TLR4 agonist, but not the response to TLR2/6 or TLR3 agonists. After confirming previous literature that the SARS-CoV-2 spike protein binds to the C-type lectin receptor DC-SIGN, they show that blocking DC-SIGN on dendritic cells appears to restore the response to LPS in DCs pre-exposed to SARS-CoV-2 spike protein or authentic virus. Finally, Raf-1 inhibition similarly restored some of the responsiveness to LPS in DCs pre-exposed to SARS-CoV-2, supporting earlier findings where activation of DC-SIGN by bacterial or viral ligands was shown to modulate TLR4 signaling via Raf-1 (e.g. Gringuis et al. (2009) Nat. Immunol.).

Overall, the manuscript presents interesting data and addresses an important topic (bacterial co-infection in the context of COVID-19). The study is carefully executed and the data are interpreted appropriately. However, it is a fairly minor conceptual advance compared to previous work. In my opinion, further mechanistic studies (see comment 1 below) would make the manuscript more suitable for publication in PLoS Pathogens.

**Part II – Major Issues: Key Experiments Required for Acceptance**
Please use this section to detail the key new experiments or modifications of existing experiments that should be absolutely required to validate study conclusions.

Generally, there should be no more than 3 such required experiments or major modifications for a "Major Revision" recommendation. If more than 3 experiments are necessary to validate the study conclusions, then you are encouraged to recommend "Reject".

Reviewer #1: (No Response)

Reviewer #2: 1. In the previous study by this group, it was shown that stimulation of DC-SIGN with HIV-1 enhanced LPS-induced cytokine expression (e.g. Gringuis et al. (2009) Nat. Immunol.), whereas here, SARS-CoV-2 dampens the LPS-induced cytokine response. What underlies this difference?

*We thank the reviewer for the comment. Although we do not know exactly what causes the different signaling between HIV-1 and SARS-CoV-2, we have now included an extensive discussion about possible explanations (lines 198-210).*

2. In Figure 3, the authors evaluate IL-8 expression in 293T/TLR4 cells, while IFN-beta, IL-6, IP-10, IL-10, etc. (and not IL-8) are evaluated in other figures. It would be good to also confirm in Figure 3 that the levels of one of the cytokines (e.g., IP-10, IL-6, IL-10) that exhibit decreased responsiveness to LPS after SARS-CoV-2 exposure in DCs is not decreased in the 293T/TLR4 cell model, to strengthen the conclusion that indeed the effect seen in DCs is not due to a direct interaction between SARS-CoV-2 and TLR4.

*We thank the reviewer for the comment. Since the TLR4-expressing HEK cell lines are different from primary DCs, they do not produce many cytokines, and IL-8 is the primary readout for TLR4-mediated activation in this cell line. After testing different cytokines, interferon responses or IL-6 were not induced by HEK cells; however, we observed production of TNF-α by these cells. We have now specified this in the text and included these data in the figure (new figure 3, lines 99-100; lines 105-108).*

3. In Figure 4A, would recommend including an isotype control antibody.

*As requested, we have now included an antibody isotype control in the DC-SIGN bead binding as well as virus binding assays (new figure 4, lines 123-124).*

**Part III – Minor Issues: Editorial and Data Presentation Modifications**
Please use this section for editorial suggestions as well as relatively minor modifications of existing data that would enhance clarity.

Reviewer #1: (No Response)

Reviewer #2: 1. Related to Figure 6, it is appreciated that the statistical analysis is clear in the figures as well as often noted in the text. The donor variance is commented upon. Even so, it is important to note in lines 136-138 that although cytokines levels appear restored with anti-DC-SIGN antibody treatment, this is only significant for IP10 and IL-6. The same goes for lines 140-143 regarding the effect of Raf-1 inhibition.

*We thank the reviewer for this comment, we now specified the significance in the text to make it clearer (lines 142-144; lines 147-149).*
